# A follow-up study of first episode major depressive disorder. Impairment in inhibition and semantic fluency—potential predictors for relapse?

**DOI:** 10.3389/fpsyg.2013.00633

**Published:** 2013-09-13

**Authors:** Marit Schmid, Åsa Hammar

**Affiliations:** ^1^Department of Biological and Medical Psychology, University of BergenBergen, Norway; ^2^Moodnet Research Group, Division of Psychiatry, Haukeland University HospitalBergen, Norway; ^3^Division of Psychiatry, Haukeland University Hospital, University of BergenBergen, Norway

**Keywords:** first-episode major depression, follow-up, executive function, inhibition, semantic fluency, relapse

## Abstract

The present study investigated the Executive Functions (EF) of inhibition, mental flexibility and phonemic and semantic fluency in a 1-year follow-up assessment of patients diagnosed with first episode Major Depressive Disorder (MDD). In the acute phase, the patient group performed significantly poorer compared to the control group (CG) in inhibition and semantic fluency. The present study pursued these findings from the acute phase to see if the impairment seen in inhibition and semantic fluency in the acute phase normalized or persisted in the follow-up assessment. In addition, the present study investigated the association between poor inhibition and semantic fluency performance and the experience of relapse during the 1-year period. Twenty eight patients and 28 individually matched control subjects were included. EF was reassessed using three tests from the Delis Kaplan Executive Function System (D-KEFS).

**Results**: There was a significant decrease in depression severity score from the acute phase, showing that most of the patients were in remission in the follow-up assessment. Results showed a sustained impairment in inhibition and semantic fluency in the patient group. However, the performance in inhibition was more severe when an additional requirement of mental flexibility was included. There were no group differences in the other EF functions measured. Further, patients with a relapse in the course of 1 year performed significantly poorer in inhibition/switching at inclusion compared to patients that did not relapse and the CG. This relationship was not found for semantic fluency. Poor performance in inhibition and semantic fluency are prolonged despite symptom reduction in patients with a first episode of MDD. Moreover, although based on a small sample of patients, the present study showed that there may be a relationship between impaired ability in the EF of inhibition/switching and vulnerability for the experience of relapse.

## Introduction

Patients with Major Depressive Disorder (MDD) are frequently found to be impaired in Executive Functions (EF) such as mental flexibility (switching), verbal fluency tasks, problem solving and planning and inhibition (see reviews Austin et al., 2001; Castaneda et al., 2008; Hammar and Årdal, 2009). However, among these findings, several studies indicate that inhibition and semantic fluency performance may be specifically impaired in this patient group. Poor inhibition and semantic fluency performance is documented in the acute phase of illness (Calev et al., 1989; Trichard et al., 1995; Fossati et al., 1999; Grant et al., 2001; Ravnkilde et al., 2002; Den Hartog et al., 2003; Fossati et al., 2003; Stordal et al., 2005; Markela-Lerenc et al., 2006; Gohier et al., 2009; Hammar et al., 2011), and has been found to persist despite symptom reduction and recovery (Paradiso et al., 1997; Reischies and Neu, 2000; Neu et al., 2005; Paelecke-Habermann et al., 2005; Smith et al., 2006; Nakano et al., 2008). Impaired inhibition and semantic fluency performance has been documented in longitudinal studies following patient groups for months and years after initial episode (Trichard et al., 1995; Biringer et al., 2005; Hammar et al., 2010; Årdal and Hammar, 2011; Schmid et al., 2011). Thus, although there is evidence of intact semantic fluency in the acute phase of illness (Austin et al., 1999) and improved inhibition in phases of remission (Merens et al., 2008), the literature in general points to a relatively firm pattern of impaired inhibition and semantic fluency in MDD. These cognitive functions may represent an enduring pattern of poor cognitive functioning in MDD, or they may show a more slowed and prolonged normalization compared to symptom severity (Årdal and Hammar, 2011; Schmid et al., 2011). It is difficult to draw conclusions based on the assumptions of possible enduring traits characterizing MDD. One reason for this is that the literature concerning the course of cognitive functioning in MDD is based on studies that have included patient groups with a history of depressive episodes (recurrent MDD). The literature is limited in knowledge concerning the course of cognitive functioning in patients who experience their first episode of MDD. Following MDD patients from their initial episode will yield valuable knowledge concerning the role of cognitive function in the course of illness.

Although limited, research on EF in first episode MDD patients has been conducted. Some of these results indicate that first-episode MDD patients may show approximately the same pattern of impairment in the acute phase as subgroups with recurrent MDD (Kaymak et al., 2010; Schmid and Hammar, 2013; see review; Lee et al., 2012). First-episode MDD patients have been found, in the acute phase of illness, to be impaired in verbal inhibition and perserverative tendency (Ilonen et al., 2000; Karabekiroglu et al., 2010). However, this subgroup of MDD patients has also been found to show impulsive behavior in decision-making and to have greater attention toward sad stimuli compared to healthy controls, but intact performance in switching attentional set (Kyte et al., 2005). Intact EF has also been reported in groups consisting of both first and recurrent MDD patients in the acute phase (Grant et al., 2001; Westheide et al., 2007). One longitudinal study including a group of first episode patients reported a general impairment across cognitive domains, including measures of EF (Reppermund et al., 2009). These findings reflect that the literature concerning EF in first-episode MDD is divergent and limited; thus, one should be cautious when interpreting EF in this patient group. In addition, little is known concerning the course of cognitive function in this patient group and the relation to depressive course of illness, thus knowledge concerning the course of EF in first-episode MDD is of interest and should be targeted in longitudinal studies.

Despite the relatively firm finding of impaired EF functions in patients with recurrent MDD, and especially impaired inhibition and semantic fluency, few researchers have tried to link the direct relationship between cognitive functioning and the experience of relapse or recurrence of symptoms. This is surprising, given that ~50% of all patients are estimated to have a relapse within 2 years, with the highest risk during the first year after initial episode (Mueller et al., 1999; Vittengl et al., 2007). During their lifetime, 80% of all patients are estimated to experience more than one episode (Hollon and Shelton, 2001; Rush, 2001). Studies have found evidence of a positive correlation between cognitive decline and number of depressive episodes (Kessing et al., 2004). However, these findings do not address the direct relationship between cognitive functioning and symptom course. One study focusing on this relationship followed first-episode and recurrent patient groups longitudinally and found that deficits in divided attention were associated with delayed response and risk to relapse (Majer et al., 2004). However, the authors have not presented data showing if there is a different trend between the first-episode and the recurrent group. This would have been interesting in order to determine whether the impairment in divided attention is dominant in the recurrent group as a result of recurrence, and not a possible predictor of relapse in patients experiencing their first episode. Another study reported an association between impaired initiation and perseveration scores and relapse and recurrence in geriatric MDD patients (Alexopoulos et al., 2000). Reppermund et al. (2009) found that cognitive impairment could not predict clinical outcome or course of MDD. However, these studies did not particularly follow patients from their initial episode. The exploration of the role cognitive functioning plays in the course of the disease should be targeted in further research. Ideally, researchers should include and follow patients from their initial episode.

The present study aimed at following a patient group diagnosed with first-episode MDD in a longitudinal perspective. The patients were included in the acute phase of illness in a study of EF in first-episode MDD (Schmid and Hammar, 2013). The D-KEFS (Delis et al., 2001), was administrated to investigate the EF of inhibition, phonemic and semantic fluency, planning and problem solving and mental flexibility (switching) in the acute phase of illness. The results from the acute phase showed that the patient group performed significantly poorer compared to the control group (CG) in the EF of inhibition, inhibition/switching and semantic fluency and in three measures relying on processing speed (naming and reading speed and visual scanning). Further, the calculation of contrast scores showed that poor processing speed could not solely account for the impaired performance in EF. In addition, there was no association between symptom severity and cognitive performance. The results from the acute phase of illness showed that impaired inhibition and semantic fluency are present initially in the course of MDD, maybe representing a stable trait independent of the previous number of depressive episodes (Schmid and Hammar, 2013).

The present study pursued findings from the acute phase of illness by retesting all subjects in a 1-year follow-up study. In addition, the study investigated the possible association between poor inhibition and semantic fluency and the tendency to experience a relapse of depression within 1 year after initial episode. The present study addressed two questions:

1. Does the poor performance in the EF of inhibition and semantic fluency seen in the acute phase of illness in first-episode MDD persist or normalize in a longitudinal perspective, independent of symptom severity scores?
2. Is there a relationship between poor inhibition and semantic fluency in the acute phase of illness and relapse of depressive symptoms within the first year after initial episode, possibly identifying different cognitive profiles in different subgroups of MDD?

Based on previous literature showing that a majority of studies find impaired inhibition and semantic fluency in groups of recurrent MDD patients, we hypothesized that the patient group would still perform poorer compared to the CG in the follow-up assessment due to either a stable cognitive impairment or a slow normalization process. Further, we hypothesized that those patients who experienced a relapse within the year following initial episode would show a more impaired performance in inhibition and semantic fluency in the acute phase of illness compared to patients that did not have a relapse.

## Methods

### Clinical and demographic data

The subjects were tested at two points in time, in the acute phase of illness (T1) and 1 year after inclusion (T2). At T1, 30 patients (16 males and 14 females) meeting the DSM-IV criteria (DSM-IV, 2000) for a unipolar first-episode MDD diagnosis, using MINI—International Psychiatric Structural Interview (Leiknes et al., 1999), were included in the study. At both test times, the structural rating scale Montgomery Åsberg Depression Rating Scale (MADRS) (Montgomery and Åsberg, 1979) was administrated to measure severity of depression.

Patients were included in the study through cooperation with doctors and psychologists in primary healthcare. Patients were given information about the present study by their doctor or psychologist. Patients deemed by their doctor or psychologist to be suitable based on the inclusion and exclusion criteria, and who consented to participate, were contacted by the study coordinator. Inclusion criteria for the patient group were a diagnosis of first-episode MDD and a minimum score of 20 on MADRS, indicating a moderate to severe depression. Patients were excluded from the study if they reported severe symptoms of depression earlier in life and if they had been diagnosed with depression and/or had received treatment for depression earlier in life. Furthermore, patients with psychosis, known brain damage, severe somatic disorders, alcohol and/or substance abuse, and patients who had been treated with electro convulsive therapy (ECT) were excluded from the study (See Table 1 for clinical and demographic variables). Three patients were excluded when recruiting patients at T1 because they met the criteria for recurrent MDD, and one patient was excluded because Norwegian was her native language and the neuropsychological tests were thereby biased because of language difficulties. Fourteen patients were prescribed antidepressant medication. The patients were outpatients, receiving either medical (13.3%), psychological (30%), or both (33.3%) for the first time, or no treatment at all (23.3%).

**Table 1:** **Descriptive data for the patient group and the control group at T2**.

**T2**	**Patient group (*N* = 28)**		**Control group (*N* = 28)**	
	***M***	***SD***	***M***	***SD***
Age	26.93	5.33	26.93	5.18
Education	14.29	1.76	14.79	1.69
Males/females	14/14		14/14	
IQ^**^	118.53	8.12	120.97	8.23
MADRS score	9.96	6.01	^*^	^*^

* Control group, no history of illness.

** IQ measured at inclusion, T1.

At T1, a CG (*N* = 30) was included, with the subjects individually matched to the patient group on the basis of gender, age and years of education (within a ±2 year limit). The CG was recruited from the University of Bergen and through acquaintances of employees of The Department of Biological and Medical Psychology of the University of Bergen. The prospective members of the control sample were interviewed to survey their history of mental or somatic disorders and were excluded if they reported a history of any mental disorder, any brain damage and/or alcohol and/or substance abuse.

All participants were asked to participate in the follow-up assessment 1 year later. At T2, data from two patients are missing due to dropout. The study coordinators were not able to regain contact with one of the patients, and one patient did not want to participate in the follow up assessment. The two individually matched control subjects were therefore not included at T2. At T2, the mean score on MADRS showed that at the follow-up assessment the patient group had minimal symptoms of depression, indicating that most patients were in a condition requiring no treatment (See Table 1 for clinical and demographic variables at T2). At T2, five patients were no longer receiving antidepressant medication, and one patient had started antidepressant medication. At T2, ten of the patients were prescribed Selective Serotonin Reuptake Inhibitors (SSRI) and one used Serotonin Noradrenaline Reuptake Inhibitor (SNRI). At T2, six patients had stopped receiving medical and/or psychological treatment, and two patients had started receiving treatment. In total, 18 patients received treatment at T2 (see Table 2 for more detailed information regarding treatment).

**Table 2:** **Descriptive data for the relapse group (RLG), the no relapse group (NRG), the no change group (NCG), and the control group (CG) at T1 and T2**.

	**RLG (*N* = 11)**		**NRG (*N* = 12)**		**NCG (*N* = 5)**		**CG (*N* = 30)**	
	***M***	***SD***	***M***	***SD***	***M***	***SD***	***M***	***SD***
**T1**								
Age	25.09	6.47	25.25	4.09	29.6	4.88	26.17	5.69
Education	14.27	1.62	14.25	1.96	13	1.41	14.03	1.65
Males/Females	3/8		10/2		1/4		14/14	
IQ	115.46	6.53	119.08	9.65	123.4	7.57	120.97	8.23
Months depressed	1.64	1.57	2.08	2.07	2	0.71	^*^	
MADRS score	25	4.36	23	2.49	28	3.16	^*^	
Treatment	Frequency/percent							
Psychological treatment.	2	18.2%	5	41.7%	1	20%	^*^	
Medical treat.	1	9.1%	1	8.3%	2	40%	^*^	
Both psych/med.	6	54.5%	4	33.3%	0		^*^	
No treatment	2	18.2%	2	16.7%	2	40%	^*^	
**T2**								
MADRS score	9.09	5.19	7.42	3.53	18	6.33	^*^	
Treatment	Frequency/percent							
Psychological treatment.	3	27.3%	1	8.3%	1	20%	^*^	
Medical treatment	1	9.1%	0		1	20%	^*^	
Both psych/med.treat.	4	36.4%	6	50%	1	20%	^*^	
No treatment	3	27.3%	5	41.7%	2	40%	^*^	

* Control group, no history of illness.

### Subgroups in MDD

At T2 all patients were interviewed retrospectively according to the course of their symptoms since inclusion to detect whether patients had experienced a relapse of their depressive illness. A drawn timeline from inclusion to follow-up was used during the interview to obtain the most accurate recall possible of the previous year's events. The definition of a relapse and remission was based on suggested operational criteria for outcomes in depression designed by Frank et al. (1991) and Rush et al. (2006). A relapse was defined as a return to a fully symptomatic state of depression after a minimum 3-week period during which minimal symptom status is maintained (remission). To fulfill the criteria of a relapse, the subject had to report the relapse period as having lasted a minimum of 2 weeks. In the present project a relapse was further defined as a period during which the subject reported difficulties performing at an optimal level in areas such as school, work or social functioning. All patients were interviewed by a psychologist.

Categorization of the patient group at T2 according to those who reported having experienced a relapse since inclusion and those who did not, resulted in three groups: a Relapse Group (RLG) (*N* = 11), a No–Relapse Group (NRG) (*N* = 12) and a group of patients who had experienced little change in symptomatology since inclusion, No Change Group (NCG) (*N* = 5). The latter group reported only minor change since inclusion and described their depression as being more chronic with short durations (days or weeks) of periods of minor symptoms. At follow-up, they have a mean MADRS score of 18, indicating a mild to moderate depression requiring treatment. The two other groups had a mean score on MADRS (>10) showing that most patients in these groups had low depression severity with no need for treatment (remission) (Hawley et al., 2002). There were no major differences between the relapse group and the no relapse group regarding treatment variables across T1 and T2. Furthermore, in the no change group, two patients had not received any treatment for their depressive symptoms across time (See Table 2 for clinical and demographic variables at T2).

### Procedure and neuropsychological assessment

The neuropsychological assessment was conducted at the Institute of Biological and Medical Psychology, University of Bergen, Norway. The testing was administered by a trained senior test technician. Due to the recruitment procedures, the test technician was not blinded to group membership for the patients and control subjects. The neuropsychological tests were given to all patients in the same sequence. The tests were part of a comprehensive test battery, including IQ measurements (WASI) and other standardized and experimental tests. All testing was performed during regular work hours and took ~4 h to complete. The procedure and tests used were the same at both test times (T1 and T2).

In the present study, performance on the Color Word Interference Test (CWIT), the Verbal Fluency Test (VFT) and the Trail Making Test (TMT) from D-KEFS was analyzed.

The CWIT comprises four conditions: Color Naming (C), Word Reading (W), Inhibition (the classic Stroop condition) (CW), and Inhibition/Switching (IS). In C and W successively, basic cognitive skills such as naming color patches and reading words are measured. In CW, the ability to inhibit the automatic response of reading is measured. In the IS, the ability to inhibit an automatic response of reading and, in addition, to shift mental set (mental flexibility) are measured.

The VFT includes three conditions: Letter Fluency (LF) (F, A, and S), Category Fluency (CF) (animals and boys' names) and Category Switching (CS) (fruit and furniture). All conditions have a maximum time limit of 60 s in which to complete each trial. All three conditions measure cognitive skills such as vocabulary knowledge, spelling ability and basic attention. For the specific conditions, cognitive functions measured are systematic retrieval of phonemically similar lexical items (LF), rapid retrieval of multiple words from a semantic category (CF), and set shifting (CS).

The TMT consists of five conditions: Visual scanning (VS), Number Sequencing (NS), Letter Sequencing (LS), Number Letter Switching (NLS) and Motor Speed (MS). Cognitive skills measured by this test are VS, basic attention, mental flexibility and MS.

Informed consent was obtained from all participants at T1. The study was performed in accordance with the Helsinki Declaration of the World Medical Association Assembly. The Regional Committee for Medical Research Ethics and The Norwegian Data Protection Authority approved the study.

### Data scoring and analyses

The statistical analysis of the data was carried out using the Statistical Package for the Social Sciences (SPSS) version 20. An alpha level of <0.05 was used for all statistical tests. Levene's test of homogeneity of variance was conducted. Data was checked for outliers. The data analyses were conducted and are reported in two main parts: First, the analysis concerning group differences between patients and controls were conducted. Second, the analysis was conducted concerning the subgroups; the relapse group (RLG), the no relapse group (NRG), the no change group (NCG) and the CG.

### Patient group and control group

Independent Samples *t*-Tests were computed to compare the groups for demographic and clinical variables at T2. To investigate the performance for the CG and the patient group across the two testing points (T1 and T2), repeated measures between-groups analysis of variance was conducted for the CWIT, VFT, and the TMT. The basic design was Group (depressed patients and control subjects) × Test occasion (Test 1 and Test 2) × Test condition. The data used were raw scores. In the CWIT and TMT, scores were measured by the number of seconds required to complete the trials. In the VFT, the scores were equivalent to the number of words produced within the time limit specified for each condition. To investigate group differences on the different test conditions at T2, multivariate analysis of variance was conducted for each test. For the TMT test, the VS condition, one of the patients was identified by SPSS as an outlier. The patient showed a major change in score from T1 to T2 with very poor performance in this condition at T2. This subject's score was excluded in the analysis of the TMT test. For the CWIT, a contrast score for inhibition (CW) and inhibition/switching (IS), for each patient and control subject, was calculated by subtracting the score in the color naming and word reading conditions from the score in the inhibition and the inhibition/switching conditions: Inhibition (CW-((C + W)/2), Inhibition/switching (IS-((C + W)/2). A multivariate analysis was then computed to investigate mean group differences based on these contrast scores. Independent Samples *t*-Tests were computed to compare the two groups on the proportion of errors made in the different conditions. Bivariate correlation was computed to investigate the relationship between cognitive function and depression severity at T2. Partial correlations were conducted to explore the relationship between scores on the inhibition condition and the category (semantic) fluency condition at T1 and T2, while controlling for depression severity measured at both test times. A multivariate—and univariate between-groups analysis of variance was conducted to investigate if there was a difference in performance in inhibition, inhibition/switching, semantic fluency and psychomotor speed between patients who used medications and those who did not.

### Subgroups and control group

A multivariate analysis of variance with post hoc comparisons (Tukey HSD) was conducted to explore the differences in cognitive functioning between the relapse group, the no relapse group, the no change group and the CG at both T1 and T2. For the inhibition condition at T2, the Levene's Test of Equality of Error Variances was not met; therefore, a significant value of 0.01 was adopted when interpreting this analysis. A paired Sample *t*-Test was administered to assess change in cognitive performance for the different subgroups from T1 to T2. Independent Samples *t*-Tests were computed to compare the subgroups in demographic and clinical variables at T1 and T2. Since there was a difference in performance between the subgroups in inhibition and inhibition/switching, a logistic regression analysis was computed using the contrast scores to explore the predictive value of poor inhibition and inhibition/switching performance in the patient group.

## Results

### Cognitive performance, patient and control group

**Figure 1:**
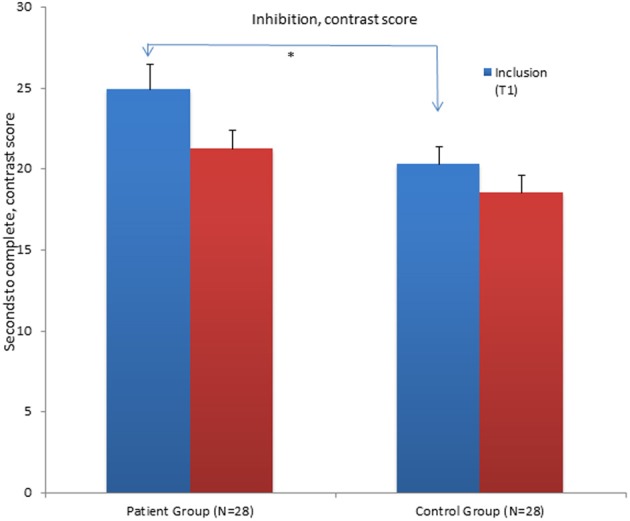
**Mean contrast scores for the Inhibition condition of the Color Word Interference Test (CWIT) for the patient group and control group at inclusion (T1) and at follow-up assessment (T2)**. ^*^Significant differences between the groups. Graphs represent mean Standard Error (SE).

**Figure 2:**
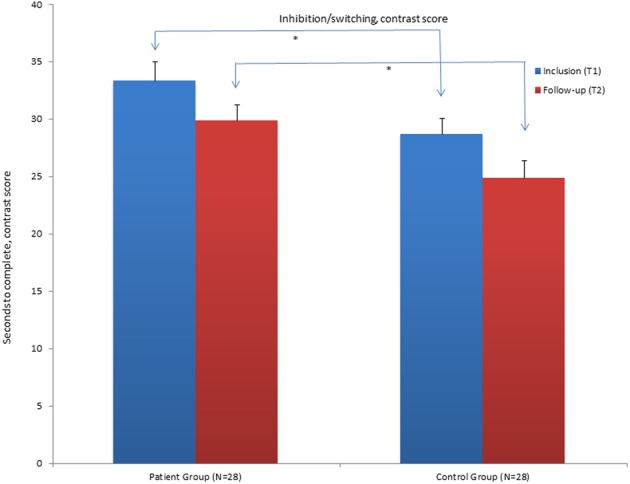
**Mean contrast scores for the Inhibition/switching condition of the Color Word Interference Test (CWIT) for the patient group and control group at inclusion (T1) and at follow-up assessment (T2)**. ^*^Significant differences between the groups. Graphs represent mean Standard Error (SE).

**Figure 3:**
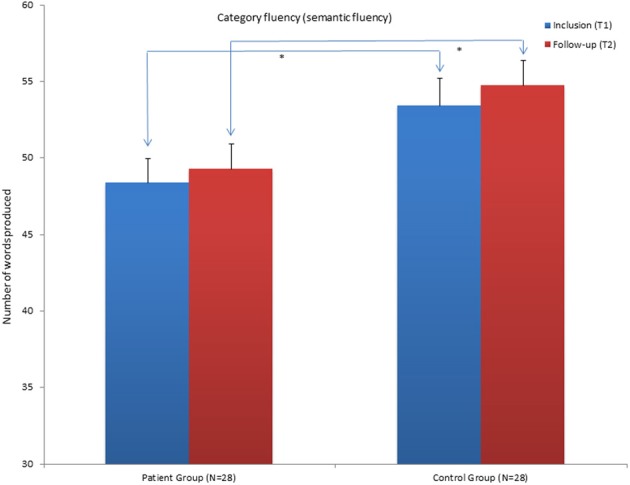
**Mean scores for the category Fluency (semantic fluency) condition of the Verbal Fluency Test (VFT) for the patient group and control group at inclusion (T1) and at follow-up assessment (T2)**. ^*^Significant differences between the groups. Graphs represent mean Standard Error (SE).

The main effects of group, time and condition were significant on the CWIT, the VFT and the TMT across T1 and T2. The interaction effect of time × group was significant in the CWIT. The interaction effects of time × condition and group × condition were significant in the CWIT and the TMT. For the VFT, no interaction effects were significant. The three-way interaction effect of time × group × condition was not significant in either test (see Table 3 and Figures 1, 2 and 3).

**Table 3:** **Cognitive performance in the patient group and control group across T1 and T2**.

		**Main effect**			**Interaction effect**			
		**Group**	**Condition**	**Time**	**Time × group**	**Time × condition**	**Group × condition**	**Time × cond. × group**
CWIT	Wilk's λ		0.041	0.58	0.92	0.605	0.856	0.936
	*F*(df)	16.05 (1, 54)	408.11 (3, 52)	39.04 (1, 54)	4.67 (1, 54)	11.33 (3, 52)	2.919 (3, 52)	1.191 (3, 52)
	Eta sq	0.23	0.96	0.42	0.08	0.4	0.14	0.064
	F-sig.	*p* < 0.000	*p* < 0.000	*p* < 0.000	*p* = 0.035	*p* < 0.000	*p* = 0.043	*p* = 0.322
VFT	Wilk's λ		0.033	0.834	0.999	0.894	0.904	0.994
	*F* (df)	4.52 (1, 54)	767.10 (2, 53)	10.78 (1, 54)	0.038 (1, 54)	3.134 (2, 53)	2.824 (2, 53)	0.153 (2, 53)
	Eta sq	0.07	0.97	0.17	0.001	0.106	0.096	0.006
	F-sig.	*p* = 0.04	*p* < 0.000	*p* = 0.002	*p* = 0.847	*p* = 0.052	*p* = 0.068	*p* = 858
TMT	Wilk's λ		0.092	0.559	0.999	0.66	0.804	0.974
	*F* (df)	5.01 (1, 53)	122.95 (4, 50)	41.77 (1, 53)	0.061 (1, 53)	6.43 (4, 50)	3.05 (4, 50)	0.338 (4, 50)
	Eta sq	0.086	0.908	0.441	0.001	0.34	0.196	0.026
	F-sig.	*p* = 0.029	*p* < 0.000	*p* < 0.000	*p* = 0.806	*p* < 0.000	*p* = 0.025	*p* = 0.851

CWIT, Color Word Interference Test; VFT, Verbal Fluency Test; TMT, Trail Making Test; df, degrees of freedom; Eta sq., Eta Squared.

The results show that the patient group still performs significantly poorer in the word reading condition, the inhibition and inhibition/switching conditions of the CWIT compared to the CG at T2. They no longer performed significantly poorer on the color naming condition. When the influence of performance in the color naming- and word-reading conditions was subtracted, the patient group still had significantly poorer performance in the contrast inhibition/switching condition (See Table 4 and Figure 2). The contrast inhibition condition did not reach statistical significance (See Table 4 and Figure 1). In the VFT, the patient group performed significantly poorer compared to the CG on the CF (semantic fluency) condition. There were no significant differences between the two groups on the LF condition and on the CS condition (See Table 4 and Figure 3). In the TMT, the patient group performed significantly poorer compared to the CG on the VS–and the NS conditions (see Table 4). There were no differences between the two groups on errors made in the different conditions of the CWIT, the VFT and the TMT.

**Table 4:** **Cognitive performance for the patient- and control group at T2**.

**TEST**	**Patient group *N* = 28**		**Control group *N* = 28**		**Stat**.		
	***M* (*SD*)**	**Change score**	***M* (*SD*)**	**Change score**	***F***	***p***	**Eta sq**.
**CWIT**							
Color naming	28.71 (3.92)	2.47	26.82 (4.04)	0.57	3.17	0.081	0.05
Word reading	22.39 (3.21)	0.4	20.04 (2.63)	−0.24	^*^9.02	0.004	0.14
Inhibition	46.82 (7.49)	5.04	41.93 (6.69)	5.04	^*^6.64	0.013	0.11
Inhibition/sw	55.43 (9.33)	4.89	48.32 (9.21)	3.93	^*^8.24	0.006	0.13
Contrast in	21.27 (5.8)	3.6	18.5 (5.69)	1.8	3.24	0.077	0.05
Contrast in/sw.	29.86 (7.34)	3.46	24.89 (8.15)	3.77	^*^5.77	0.02	0.1
**VFT**							
Letter fl.	48.43 (12.28)	−3.47	52.82 (12.74)	−3.89	1.73	0.194	0.03
Category fl.	49.25 (9.54)	−0.86	54.75 (8.6)	−1.36	^*^5.13	0.028	0.08
Category sw.	15.25 (2.65)	−0.97	15.89 (2.44)	−0.5	0.89	0.349	0.02
TMT	(*N* = 27)						
Visual scan.	17.93 (2.80)	1.11	15.86 (3.57)	0.5	^*^5.70	0.021	0.09
Number seq.	24.30 (8.20)	4.45	19.0 (5.75)	5.46	^*^7.73	0.008	0.127
Letter seq.	21.48 (6.04)	4.59	20.21 (5.32)	4.15	0.68	0.413	0.013
Numb-let.seq.	62.40 (17.24)	9.52	53.43 (18.39)	9.64	3.48	0.068	0.062
Motor speed	17.22 (5.49)	2.89	17.68 (6.51)	1.15	0.08	0.780	0.001

Mean (M), standard deviation (SD) and change score (raw score) from T1 to T2. Statistics (F, p, Eta sq.) on differences between groups at T2.

* Significant differences between groups at T2.

For the patient group there was a significant positive partial correlation between scores on the contrast inhibition condition at T1 and at T2, while controlling for depression severity measured by MADRS at both T1 and T2, *r* = 0.645, *n* = 24, *p* < 0.001. This pattern was also true for the contrast inhibition/switching condition; *r* = 0.392, *n* = 24, *p* < 0.047, the semantic fluency condition; *r* = 0.757, *n* = 24, *p* < 0.001, and the VS condition; *r* = 0.648, *n* = 24, *p* < 0.001. Impaired performance in the acute phase (T1) was associated with impaired performance at follow-up assessment (T2), independently of symptom severity at both test times.

### Clinical and demographic data, patient and control group

There were no significant differences between the patient group and the CG in demographic variables at T2 (see Table 1). In terms of medication use, there were no significant differences between patients who used medication and those who did not on the conditions of the CWIT and on the VS condition of the TMT. There were no significant differences between the groups in the four conditions of the CWIT; *F*_(4, 23)_ = 0.554, *p* < 0.698; Wilks' Lambda = 0.912, partial η^2^ = 0.08. Fur thermore, there were no significant differences between the groups in the two contrast scores of inhibition and inhibition/switching; *F*_(2, 25)_ = 0.183, *p* < 0.834; Wilks' Lambda = 0.986, partial η^2^ = 0.014. No differences were detected between the patient group that did not use medication (*M* = 18.12, *SD* = 2.87) and the group that did use medication (*M* = 19.00, *SD* = 5.35) on the VS condition; *F*_(1, 5.20)_ = 0. 324, *p* < 0.574, partial η^2^ = 0.012. However, the group that used medication (*M* = 28.45, *SD* = 10.42) performed significantly poorer compared to the group that did not use medications (*M* = 21.94, *SD* = 5.08) on the NS condition of the TMT, *F*_(1, 283.33)_ = 4.912, *p* < 0.036, partial η^2^ = 0.16. Furthermore, on the VFT the patients who used medication (*M* = 53.81, *SD* = 10.36) performed significantly better than those patients who did not use medication (*M* = 46.29, *SD* = 7.93) on the CF (semantic fluency) condition, *F*_(1, 378, 1)_ = 4.73, *p* < 0.039, partial η^2^ = 0.15.

There was a significant decrease in depression severity score measured by MADRS from T1 (*M* = 24.68, *SD* = 3.79) to T2 (*M* = 9.96, *SD* = 6.01), *t*_(27)_ = 13.96, *p* < 0.001 (two-tailed). The mean decrease in MADRS was 14.71 with a 95% confidence interval ranging from 12.55 to 16.88 (See Table 1) There was no significant correlation between depression severity and cognitive performance at T2 for the word reading condition of the CWIT, *r* = 0.076, *N* = 28, *p* < 0.702, the inhibition condition of the CWIT, *r* = −0.056, *N* = 28, *p* < 0.777, the inhibition/switching condition of the CWIT, *r* = −0.027, *N* = 28, *p* < 0.890, the contrast score in inhibition, *r* = −0.171, *N* = 28, *p* < 0.384, or the contrast score of inhibition/switching, *r* = −0.113, *N* = 28, *p* < 0.567. Furthermore, there was no significant correlation between depression severity and performance in the category of fluency (semantic fluency) of the VFT, *r* = −0.075, *N* = 28, *p* < 0.703, the VS condition of the TMT, *r* = 0.343, *N* = 28, *p* < 0.074, or the NS condition of the TMT, *r* = 0.069, *N* = 28, *p* < 0.726.

### Cognitive function, subgroups and control group

#### Color word interference test

The results showed that there were differences between the four groups concerning performance at T1. Pursuing the main group effect on the combined dependent variables on the CWIT, *F*_(12, 129.93)_ = 2.93, *p* < 0.001; Wilks' Lambda = 0.530, partial η^2^ = 0.19, *post-hoc* comparisons using the Tukey HSD test showed that the relapse group performed significantly poorer compared to the CG on all conditions of the CWIT at T1. On the inhibition/switching condition, the relapse group was significantly poorer compared to the no relapse group and the CG. Following the main group effect on the contrast scores of inhibition and inhibition/switching, *F*_(6, 102)_ = 2.498, *p* < 0.027; Wilks' Lambda = 0.760, partial η^2^ = 0.13, *post-hoc* comparisons showed that the relapse group was significantly poorer compared to the no relapse group and the CG on the contrast inhibition/switching condition at initial testing (T1) (See Table 5 for Mean scores and Standard Deviations).

**Table 5:** **Cognitive performance in the relapse group (RLG), the no-relapse group (NRG), the no change group (NCG) and the control group (CG) at T1 and T2**.

	**RLG, *N* = 11**		**NRG, N = 12**		**NCG, *N* = 5**		**CG, *N* = 28**		**Stat**.	
	***M***	***SD***	***M***	***SD***	***M***	***SD***	***M***	***SD***	**Sig**.	***Post-hoc***
**T1-INITIAL TEST**										
**CWIT**										
Color na.C	33.1	3.86	30.42	3.23	28.8	3.49	27.39	3.57	^*^	RLG < CG, NRG, NCG
Word re.W	24.27	3.2	22.33	2.81	20.6	2.61	19.79	2.83	^*^	RLG < CG, NRG, NCG
Inhib.CW	54.64	9.3	50.67	9.61	48.6	10.36	43.89	6.42	^*^	RLG < CG, NRG, NCG
Inhib/sw.IS	66.91	9.2	55.5	8.17	57.4	7.95	52.25	8.77	^*^	RLG < CG<NRG, NCG
Contrast CW	25.96	7.04	24.29	9.81	23.9	8.29	20.3	5.83	NS	RLG, CG, NRG, NCG
Contrast IS	38.23	8.57	29.13	8.45	32.7	7.22	28.66	7.45	^*^	RLG < CG < NRG, NCG
**VFT**										
Letter fl.LF	46.27	10.86	43.25	10.77	46.2	15.22	48.93	12.37	NS	RLG, CG, NRG, NCG
Cat.fl.CF	48.64	10	46.42	7.91	52.6	4.39	53.39	8.86	NS	RLG, CG, NRG, NCG
Cat.sw.CS	14.36	2.54	14.5	3.18	14.6	2.7	15.39	1.99	NS	RLG, CG, NRG, NCG
TMT										RLG, CG, NRG, NCG
Visu.scan.VS	18.91	4.93	19.75	3.67	18.2	3.96	16.36	4.09	NS	RLG, CG, NRG, NCG
Num.seq.NS	28.63	7.41	30.25	11.72	24.2	4.6	24.46	6.55	NS	RLG, CG, NRG, NCG
Letter.seq.LS	26.46	7.08	26.67	7.05	22.4	6.11	24.36	7.75	NS	RLG, CG, NRG, NCG
Nu/le.seq.NLS	69.09	16.16	71.17	19.65	66.6	19.54	63.07	20.18	NS	RLG, CG, NRG, NCG
Mot.speed.MS	21.46	6.85	18.17	5.89	20.8	7.4	18.82	6.52	NS	RLG, CG, NRG, NCG
**T2-FOLLOW UP ASSESMENT**										
**CWIT**										
Color na.C	30.46	4.37	27.25	2.77	28.4	4.51	26.82	4.04	NS	RLG, CG, NRG, NCG
Word re.W	23.27	2.65	21.92	2.64	21.6	5.41	20.04	2.63	^**^	RLG < CG, NRG, NCG
Inhib.CW	49.64	6.44	45.5	4.4	43.8	13.55	41.93	6.69	^**^	RLG < CG, NRG, NCG
Inhib/sw.IS	60.54	7.29	51.42	7.56	53.8	13.16	48.32	9.21	^*^	RLG < CG, NRG, NCG
Contrast CW	22.77	5.49	20.92	4.24	18.8	9.41	18.5	5.69	NS	RLG, CG, NRG, NCG
Contrast IS	33.68	5.24	26.83	7.11	28.8	9.34	24.89	8.15	^*^	RLG < CG, NRG, NCG
**VFT**										
Letter fl.LF	50.91	12.99	45.67	13.02	49.6	9.4	52.82	12.74	NS	RLG, CG, NRG, NCG
Cat.fl.CF	48.64	10.71	49.5	8.89	50	10.42	54.75	8.61	NS	RLG, CG, NRG, NCG
Cat.sw.CS	16.27	3.26	14.5	2.58	15.8	1.79	15.89	2.44	NS	RLG, CG, NRG, NCG
TMT	RLG, *N* = 11		NRG, *N* = 12		NCG, *N* = 4		CG, *N* = 28			
Visu.scan.VS	17	2.79	17.92	2.54	20.5	2.526	15.86	3.57	^***^	RLG, NRG, CG > NCG
Num.seq.NS	25.18	10.61	24.67	6.92	22.75	3.59	19	5.75	NS	RLG, CG, NRG, NCG
Letter.seq.LS	21	4.45	22.58	7.57	19.5	5.45	20.21	5.32	NS	RLG, CG, NRG, NCG
Nu/le.seq.NLS	63.27	11.87	64.25	19.19	54.5	25.63	53.43	18.38	NS	RLG, CG, NRG, NCG
Mot.speed.MS	18.73	5.58	15.25	4.45	19	7.53	17.68	6.52	NS	RLG, CG, NRG, NCG

Mean (M), standard devation (SD) and statistics (Sig., Post-hoc).

* Significant at the p < 0.01.

** Significant at the p < 0.025.

*** Significant at the p < 0.05; NS, no sig.differences between groups.

**Figure 4:**
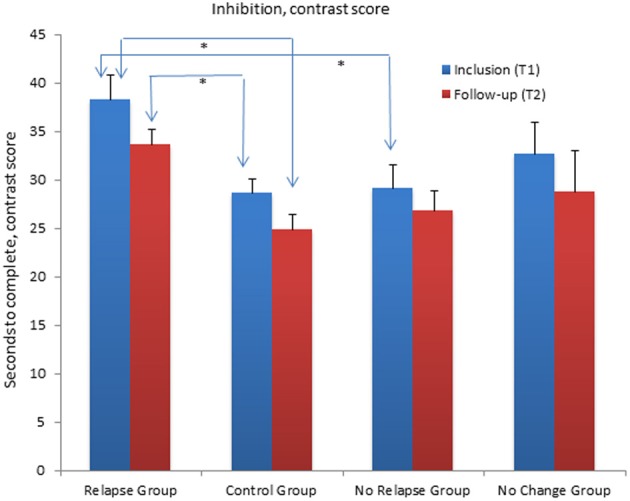
**Mean contrast scores for the Inhibition/switching condition of the Color Word Interference Test (CWIT) for the relapse group, the no relapse group, the no change group and the control group at inclusion (T1) and at follow-up assessment (T2)**. The Relapse Group performed significantly poorer compared (*p* < 0.01) to the No Relapse Group and the Control Group at T1. At T2 the Relapse group performed significantly poorer than the Control Group (*p* < 0.01). Graphs represent mean Standard Error (SE).

Following the results from T1 showing that the relapse group performed significantly poorer compared to the CG on the color naming, the word reading, inhibition and inhibition/switching conditions and additionally poorer compared to the no relapse group on the contrast inhibition/switching condition, *post-hoc* comparisons showed that the relapse group exhibits almost the same pattern of impairment in the follow-up assessment (T2). However, the relapse group did not differ significantly from the no relapse group on these conditions at T2. No other significant differences between subgroups on the CWIT were identified at T1 or T2 (see Table 5 for mean scores and Standard deviations and Figure 4).

#### Change in cognitive performance from T1 to T2 in the CWIT

The relapse group, *t*_(10)_ = 3.04, *p* < 0.012 (two-tailed) and the no relapse group, *t*_(11)_ = 5.27, *p* < 0.001 (two-tailed), showed a significant improvement in mean score on the word reading condition. The no relapse group showed an additional significant improvement in the inhibition condition, *t*_(11)_ = 2.81, *p* < 0.017 (two-tailed). The CG showed a significant improvement on the inhibition/switching condition, *t*_(27)_ = 3.34, *p* < 0.002 (two-tailed) and the contrast inhibition/switching condition, *t*_(27)_ = 3.20, *p* < 0.004. The no change group did not show any significant improvement from T1 to T2 (see Table 5 for Mean scores and Standard Deviations and Figure 4).

#### Verbal fluency test

At T1 and T2, the main effect of differences between groups was not significant. Furthermore, there were no significant differences between the four groups when considering the variables independently (see Table 5 for Mean scores and Standard Deviations).

#### Change in cognitive performance from T1 to T2

The relapse group showed a significant improvement on the LF condition, *t*_(10)_ = −2.42, *p* < 0.036 (two-tailed), and on the CS condition, *t*_(10)_ = −2.35, *p* < 0.041 (two-tailed). The CG showed a significant improvement on the LF condition, *t*_(27)_ = −2.15, *p* < 0.041 (two-tailed). The no change group and the no relapse group did not show any significant improvements from T1 to T2 (see Table 5 for Mean scores and Standard Deviations).

#### Trail making test

There were no differences between the groups at T1. Pursuing the main group effect at T2, *F*_(15, 130.15)_ = 1.99, *p* < 0.020; Wilks' Lambda = 0.564, partial η^2^ = 0.17, *post-hoc* comparisons showed that the no change group was significantly poorer compared to the CG in the VS condition. There were no differences between the other groups on the TMT at T2 (see Table 5 for Mean scores and Standard Deviations).

#### Change in cognitive performance from T1 to T2

The relapse group showed a significant improvement on the LS condition, *t*_(10)_ = 2.96, *p* < 0.014 (two-tailed). The no relapse group showed a significant improvement on the VS condition, *t*_(11)_ = 2.25, *p* < 0.046 (two-tailed), the LS condition, *t*_(11)_ = 2.35, *p* < 0.039 (two-tailed) and the MS condition, *t*_(11)_ = 3.09, *p* < 0.010 (two-tailed). The no change group did not show significant changes across time. The CG showed significant improvement on the NS condition, *t*_(27)_ = 5.43, *p* < 0.001 (two-tailed), the LS condition, *t*_(27)_ = 2.71, *p* < 0.012 (two-tailed) and the number-letter switching condition, *t*_(27)_ = 4.55, *p* < 0.001 (two-tailed) (see Table 5 for Mean scores and Standard Deviations).

### Clinical and demographic data, subgroups and control group

There were no significant differences in age, IQ and years of education between the relapse group, the no relapse group and the CG at T1 and T2. Furthermore, there was no difference between the relapse group and the no relapse group in months depressed and/or depression severity at T1 and T2. The no change group showed significantly higher MADRS score at T2, *t*_(14)_ = −2.98, *p* < 0.010 (two-tailed), and a significantly higher mean score in IQ, *t*_(14)_ = −2.15, *p* < 0.049 (two-tailed) compared to the relapse group. The no change group showed significantly higher MADRS score at T1, *t*_(15)_ = 3.50, *p* < 0.003 (two-tailed), and T2, *t*_(15)_ = 4.47, *p* < 0.001 (two-tailed) compared to the no relapse group, *t*_(14)_ = −2.98, *p* < 0.010 (two-tailed). There was a difference between the three subgroups regarding the distribution of males and females, with a substantial portion of females in the relapse group compared to the opposite pattern in the no relapse group (see Table 2).

### The relationship between relapse and cognitive function

Direct logistic regression was performed to assess the impact of poor performance in inhibition and inhibition/switching on the likelihood of experiencing a relapse. The full model containing the predictors (contrast score inhibition and inhibition/switching) was significant, χ^2^_(2, *N* = 28)_ = 6.208, *p* < 0.045, indicating that the model was able to distinguish between patients who reported having had a relapse and those who did not. The model as a whole explained between 19.9% (Cox and Snell R square) and 26.9% (Nagelkerke R squared) of the variance, and correctly classified 64.3%. Performance in inhibition/switching was the only significant contribution to the model, with an odds ratio of 1.146. This indicated that patients who show poor performance in inhibition/switching in the acute phase of illness were 1.146 times more likely to experience a relapse within the first year (see Table 6).

**Table 6:** **Logistic regression predicting the effect of poor inhibition and inhib/switching performance on tendency to relapse**.

	***B***	***SE***	**Wald**	***df***	***p***	**Odds *R***	**95% CI for odds ratio**
							**Lower/Upper**
Contr.s inhibition	−0.011	0.055	0.036	1	0.849	0.99	0.888/1.102
Contr.s. inhib/sw.	0.137	0.067	4.141	1	0.042	1.146	1.005/1.307
Constant	−4.797	2.41	3.961	1	0.047	0.008	

## Discussion

The results from the present study supported the first hypothesis of persistent cognitive impairment following the first year from initial episode in a group of first-episode unipolar MDD patients. The results show that the patient group shows a prolonged impairment in the EF functions of inhibition, inhibition/switching and semantic fluency compared to the CG, despite significant symptom reduction. More specifically in terms of the inhibition measure, the results indicate that the patient group experiences more difficulties when both inhibition and mental flexibility are requirements for completing the task. The patient group did not differ significantly from the CG in performance on the other EF measures administrated, such as mental flexibility across EF and phonemic fluency. Furthermore, following the almost identical pattern from the acute phase, the patient group performed significantly poorer compared to the CG in three conditions that rely on processing speed: the word naming condition, the VS, and NS conditions. However, the results showed that poor processing speed could not solely account for the poor performance in EF.

The results further showed that there may be different subgroups in MDD which show a different course of illness and different cognitive profiles. According to clinical and demographic data, the three groups showed a difference in severity of depression, with the no change group having a significant higher mean severity score at both T1 and T2. The no change group also had a significantly higher IQ score compared to the two other groups. In addition, there was an unequal gender distribution, with a substantial number of women in the relapse group. The three groups and the CG did not differ concerning other demographic and clinical variables. Concerning cognitive functioning, our second hypothesis was partly supported. Although based on small samples, the present study found a tendency for those patients who experienced a relapse within the first year after initial episode to perform poorer in the EF of inhibition/switching compared to those who did not experience relapse and the CG. This pattern was not evident for the semantic fluency condition.

The findings in the present study showing that the patient group in general had sustained impairment in inhibition, inhibition/switching and semantic fluency confirm previous findings of prolonged impairment in inhibition and semantic fluency in patients with recurrent MDD (Trichard et al., 1995; Biringer et al., 2005; Hammar et al., 2010; Årdal and Hammar, 2011; Schmid et al., 2011). The present results indicate that impairments in these EF may normalize later than symptoms of depression or that the deficits represent stable traits in MDD that is visible from initial episode. This conclusion is supported by reviews and meta-analysis which postulates that EF impairment seems to be a stable cognitive deficit in MDD (Douglas and Porter, 2009; Lee et al., 2012; Snyder, 2012). Especially for the EF function of inhibition with an extra demand of mental flexibility, the present study indicates that this function may represent a cognitive vulnerability factor that may contribute to relapse in some individuals. However, since this finding is based on a small sample of subjects, and the supposition that there may be other variables that may account for such a development, these findings need to be replicated in order to establish firmer interpretations.

The intact performance in the EF of phonemic fluency has been reported by previous studies in the literature (see review Henry and Crawford, 2005). The finding of different performances in these two verbal fluency measures may be explained by the hypothesis that phonemic and semantic fluency performance relies on different retrieval processes (Henry and Crawford, 2005). It has been suggested that phonemic fluency performance is dependent on search strategies based primarily on lexical representations, while semantic fluency requires searching for semantic targets in memory (Henry and Crawford, 2005). Regarding the EF of mental flexibility, this function is often reported to be impaired in MDD (Snyder, 2012; Lee et al., 2012). Furthermore, mental flexibility has been found to be more impaired in patients who have experienced a relapse (Majer et al., 2004). In the present study, the EF of mental flexibility was impaired when the additional ability to inhibit was required, and not an impairment seen across the EF measures included. Thus, the present study indicates that the ability to inhibit should not be underestimated when trying to identify possible factors influencing the susceptibility to relapse.

The possible effect of prolonged impairment in some measures of processing speed are important to further discuss, given that several researchers find MDD patients to be impaired on these measures, especially in the acute phase of illness (Tsourtos et al., 2002; Den Hartog et al., 2003; Egeland et al., 2003, 2005; Lee et al., 2012). Poor processing speed in MDD has also been found to be sensitive to clinical state (see review Douglas and Porter, 2009; Lee et al., 2012). The significant improvement on some measures of processing speed and measures of EF in the relapse and the no relapse group could be related to symptom decline and treatment. However, although findings indicate that the No Change Group performed poorly on one measure of processing speed in the follow-up assessment and that this subgroup did not show the same improvement in cognitive functioning as the two other groups across time, there were no other indications of symptom severity affecting cognitive performance in the present study. For the No Change Group in particular, however, the finding of significant higher mean score in IQ may have affected this relationship. High IQ in this group may have had impact on their cognitive performance. Furthermore, the finding of prolonged impairment in some measures of processing speed in the patient group in general at the follow-up assessment indicates that first-episode MDD patients group may still struggle with impaired performance in processing speed despite symptom decline.

Regarding symptom severity in a longitudinal perspective, the patient group as a whole showed a significant reduction in symptom severity, indicating a phase of remission at T2. However, it is important to highlight the finding of higher illness severity scores in the no change group at both T1 and T2, possible reflecting a different course of illness compared to the two other groups. However, there is not enough evidence in the present study to conclude upon this assumption. The subgroups in the present study are too small and the results are too premature to draw firm conclusions regarding these questions. Therefore, future studies should replicate these findings and include larger subgroups.

Another important finding was the unequal distribution of gender in the different subgroups. Of the patients who experienced a relapse, 72.7% were women, indicating that gender may be a factor to consider regarding susceptibility to experiencing a relapse of depression. The effect of gender on the development of recurrence in depression is controversial and provides no clear-cut findings (see review Piccinelli and Wilkinson, 2000). Furthermore, the effect of gender on cognitive performance in MDD has not received much attention in the literature (see review; Porter et al., 2007); thus, little is known about gender differences across EF. One probable reason for this is the fact that few studies have included a comparable number of men and women in their studies.

It is important to discuss the result showing that there was no relationship between poor performance in semantic fluency and the tendency to experience relapse within 1 year after initial episode. The four subgroups did not differ with regard to performance in semantic fluency. The prolonged impaired performance in semantic fluency seen in the depressed group as a whole may therefore not represent a factor that influences the course of the disease, but may be important for other characteristics of the disorder. This is a somewhat surprising finding, given that that impaired semantic fluency is a relatively firm finding in recurrent MDD (Henry and Crawford, 2005) and often reported to coexist with impaired inhibition (Biringer et al., 2005; Nakano et al., 2008; Schmid et al., 2011; Schmid and Hammar, 2013). Contrary to this pattern of coexistence, Trichard et al. (1995) semantic fluency performance has been found to normalize in accordance with symptom decline, while performance in inhibition remains impaired. In the present study, there was evidence that those patients received medical treatment to perform better in semantic fluency, and this may support the notion of apparent normalization. However, there is no evidence to conclude upon this assumption, given that the patient group in general is still impaired on the EF despite symptom reduction and treatment.

Thus, the question of why MDD patients perform poorly in inhibition and semantic fluency is important to address. It has been suggested that semantic fluency performance is dependent on semantic memory and retrieval strategies (Henry and Crawford, 2005; Neu et al., 2005) thus affecting the individual's ability to retrieve words efficiently. The ability to inhibit is postulated as representing the cognitive process controlling the individual's processing of internal and external stimuli (Joormann et al., 2007; Gohier et al., 2009), thus suggesting that this cognitive function is vital to the understanding of the cognitive deficits often reported in MDD. Inability to inhibit is reported to be correlated to the patient's sustained rumination with negative information (see review Joorman and Gotlib, 2010). Further, ruminative thoughts have been associated with susceptibility to the development and recurrence of depression (Nolen-Hoeksema, 2000). The present findings support the suggestion that the inability to inhibit may be a core cognitive function that can explain many of the challenges reported by MDD patients. However, the results indicate that inhibition alone does not add to the predictive value of the experience of a relapse of depression, but rather that the ability to inhibit is made more difficult when additional mental flexibility is needed. This interaction of inhibition and mental flexibility should be targeted in future studies that investigate traits that may influence vulnerability to relapse in MDD, especially since both cognitive functions seem to be important in this respect. Future studies should also focus on investigating the processes that govern poor performance in inhibition and semantic fluency in MDD. The results indicate that these two cognitive constructs may manifest themselves differently and are governed by different factors constituting MDD, which will have implications for the future understanding of cognitive impairment in MDD.

In sum, the present results show that impairment in the EF of inhibition, inhibition/switching and semantic fluency is prolonged despite symptom reduction in a group of first-episode MDD patients in a 1-year follow-up assessment. The results further indicate that different subgroups of MDD may have different clinical and cognitive profiles. Although based on small samples, the present study indicates that patients who are more impaired in their ability to inhibit, especially when the additional demand of mental flexibility is required, may be more vulnerable to experience a relapse of their illness during the first year after initial episode. However, to our knowledge, this study is the first that exclusively follows first-episode MDD patients longitudinally. Therefore, more studies are needed in order to draw firm conclusions regarding these issues.

### Methodological considerations

The present study contributes in several ways to the existing literature concerning EF in MDD. First, the present study has included and longitudinally investigated a patient group diagnosed with first-episode MDD. This design offered the opportunity to leave out the possible effect of previous episodes of MDD at initial inclusion and to investigate the relationship between EF functioning and symptom course. Second, the present study included a CG matched individually to the patient group, ruling out the effect of learning across time periods. However, there are important issues concerning the methodology that need to be addressed. The study included a relatively young, outpatient population. In addition, the sample showed few or no comorbid disorders. These factors are considered to be strengths of the present study since the effects of age, comorbidity and hospitalization have been shown to affect cognitive performance (see review: Snyder, 2012). However, caution should be taken when generalizing the results to other patient groups. Another important issue to discuss is the method used when identifying patients' course of illness. At the follow-up assessment, the MINI—International Psychiatric Structural Interview (Leiknes et al., 1999) was not administered. Although the patients were screened for depression severity and symptoms and were interviewed according to their course of illness, important information about potential comorbid disorders may have been lost. Furthermore, the study included thirty patients comprising a group divided into three subgroups based on relapse experience. Thus, due to low N, the results could have been affected by relatively low power. Furthermore, antidepressant medication was prescribed for eleven patients at the follow-up assessment. The effects of modern antidepressant medication such as SSRI and SNRI are not fully understood, but are recognized as being minimal (Biringer et al., 2009). In the present study there was evidence that patients who used medication performed significantly poorer compared to those who did not use medication on the NS condition of the TMT, indicating that medication may affect psychomotor speed. The opposite pattern was evident for the category (semantic) fluency condition. Those who were treated with antidepressant medication performed better compared to those who did not use medication on the semantic fluency test, a pattern which may be indicative of medication having a positive effect on this EF. An enhancement in cognitive performance due to the use of antidepressant medication has been found in studies on emotional information processing (Harmer et al., 2009). Although this relationship was not found for the other EF measures included in the present study, it may be important to further pursue this perspective in future studies.

The strength and the limitations mentioned here point to the importance of replicating the present study. In particular, studies should focus on investigating EF in the course of MDD and include a larger sample of subjects in each subgroup.

### Clinical implications

The recurrent and chronic nature of MDD represents a major challenge for the subject experiencing depression and for therapeutic interventions aimed at preventing this development. Knowledge concerning prolonged cognitive impairment in this patient group and the possible predictors of relapse will contribute toward identifying intervention strategies that should be applied and will aid in identifying patients prone to experiencing a relapse of depressive symptoms.

## Funding

The present study was funded by the Research Council of Norway (NFR); Helse Vest and the University of Bergen. The funding institutions did not participate in the collection, analysis and interpretation of the data, in the writing of the report or in the decision to submit the paper for publication.

### Conflict of interest statement

The authors declare that the research was conducted in the absence of any commercial or financial relationships that could be construed as a potential conflict of interest.
